# Supplementary material for: Enhanced magnetic spin–spin interactions observed between porphyrazine derivatives on Au(111)
Source: Commun Chem. 2020 Mar 20;3:36. doi: 10.1038/s42004-020-0282-5 (PMC9814269; doi:10.1038/s42004-020-0282-5)
Supplement: Supplementary file 2 — Supplementary Information [file 42004_2020_282_MOESM2_ESM.pdf]

Supplementary Information

Enhanced Magnetic Spin-Spin Interactions Observed between Porphyrazine-Derivatives on Au(111)

Jie Hou *et al*.

Supplementary Note 1. Atomic coordinates of the optimized structure of phase I film of VOTTDPz molecules, which are the results of structural optimization with VASP. The coordinates of gold substrate is included. The coordinates are expressed with a POSCAR/CONTCAR format used in VASP calculation.

```
project
   1.00000000000000
    11.5199999999999996    0.0000000000000000    0.0000000000000000
    12.9600000000000009   22.4499999999999993    0.0000000000000000
     0.0000000000000000    0.0000000000000000   25.0000000000000000
C N O S V
  32   32    2    8    2 108
Selective dynamics
Direct
  0.7088983716892593  0.5793057555143875  1.0036989304478201   T   T   T
  0.9139246686421746  0.1298638301891076  1.0208740135148773   T   T   T
  0.7303571838767248  0.4343997380814798  1.0239580985358336   T   T   T
  0.6006811783651576  0.1270492630596871  1.0441329325881612   T   T   T
  0.7635358371284240  0.4803924791966379  1.0105218378904928   T   T   T
  0.7381046532122908  0.1101601508272972  1.0308943755718181   T   T   T
  0.6885759419544671  0.6726617238606176  1.0084543543015179   T   T   T
  0.1064289314656020  0.1357127621593221  1.0064563910255855   T   T   T
  0.0466765049077448  0.6004269216962279  0.0347765684714244   T   T   T
  0.2887060801157367  0.9716922808983676  0.0169177397293498   T   T   T
  0.1528901125464278  0.6144647820019324  0.0401150310807107   T   T   T
  0.4194248240682253  0.9207990858108777  0.0307022332571971   T   T   T
  0.2752217428750784  0.5637893043520129  0.0232376458497584   T   T   T
  0.3950770197307346  0.8741403052848927  0.0472717969754799   T   T   T
  0.2446010233237019  0.5159380526579863  0.0146006785276452   T   T   T
  0.2567639381682038  0.8927849077595207  0.0347353585470319   T   T   T
  0.8542516842723202  0.3818130659965450  1.0362410162873255   T   T   T
  0.5760965835809321  0.0819977140210902  1.0226975323872716   T   T   T
  0.9623130215037573  0.3936064882995210  1.0246161728613994   T   T   T
  0.7067835516309181  0.0325094197582788  1.0065188730270807   T   T   T
  0.1854961390275690  0.3663402830008564  1.0137436400534525   T   T   T
  0.8550707017394457  0.9302282032441717  1.0057716498868874   T   T   T
  0.3246003122343220  0.3227038758024660  1.0043187628806027   T   T   T
  0.8896478417498100  0.8677651705692238  1.0056132363818118   T   T   T
  0.3971466773207415  0.3540603515722479  1.0000565613131871   T   T   T
  0.0310406970802100  0.8333893338764170  1.0150021302602077   T   T   T
  0.8241893340262771  0.6281730552452953  1.0220249472926140   T   T   T
  0.1419955536146429  0.0733868622626831  1.0103664552874545   T   T   T
  0.6161652000854647  0.6424850017029077  0.9969509069456369   T   T   T
 -0.0363977555032476  0.1705819453646673  1.0126736974984232   T   T   T
  0.3012664401356301  0.4169199426305069  1.0060725656747624   T   T   T
  0.0812901028921595  0.8739872617319047  1.0213296217553129   T   T   T
  0.6748242829218134  0.5371933387548277  1.0021679292936823   T   T   T
  0.7876225981280233  0.1465427824513708  1.0329054184555218   T   T   T
  0.0909091935962275  0.3522065699765409  1.0226842296562373   T   T   T
  0.7316755748796302  0.9767740467431948  1.0006851670719503   T   T   T
  0.1739506112323604  0.4227949521893312  1.0148994433023510   T   T   T
  0.9742658994984664  0.9319738758942233  1.0155665014262705   T   T   T
  0.3341573608467117  0.4594703711377912  0.0056173012270191   T   T   T
```

0.2079228115239815	0.8560850253010667	0.0339321452578833	T	T	T
0.5308893337549961	0.3189815589408072	0.9924814993932977	T	T	T
0.0834405043185385	0.7746226177621101	1.0193876055592990	T	T	T
0.3983048733724577	0.2631177201011503	1.0011087953577249	T	T	T
0.8300588729426152	0.8344790846270568	1.0018494408053955	T	T	T
0.9047789710065055	0.4545405231751903	1.0127334510675625	T	T	T
0.8020897215678734	0.0514872278507801	1.0112093651790277	T	T	T
0.8431526940079584	0.3369898743140107	0.0575028065663396	T	T	T
0.4516238460100452	0.0913928145208786	0.0309267562476750	T	T	T
0.6199403398573970	0.4305802442132440	0.0340320368223412	T	T	T
0.4994010477541393	0.1699262793683295	0.0726246306767146	T	T	T
0.4865594481271323	0.6789722652241547	0.9820872538676552	T	T	T
0.9106027844383412	0.2296963376828114	1.0120158523658347	T	T	T
0.6200275988794870	0.7321027302068204	1.0044872126512259	T	T	T
0.1666976894003271	0.1685717656407163	1.0006999408318038	T	T	T
0.8347558348348582	0.5722899588655991	1.0186697624391117	T	T	T
0.0223740206888976	0.0718894409644978	1.0180466442300344	T	T	T
0.9179897817853211	0.6417380508559853	0.0343235470977007	T	T	T
0.2660345836901228	0.0267172514391286	0.0099244462608672	T	T	T
0.1600728759548537	0.6629668800400916	0.0523024112241026	T	T	T
0.5468605220574749	0.9084996503776380	0.0364409346682651	T	T	T
0.3806195246933532	0.5728477918875630	1.0206178419966925	T	T	T
0.4997571579037969	0.8265804229575093	1.0696263621992745	T	T	T
0.1050112107753070	0.5398615660969516	1.0218548817299791	T	T	T
0.1923657902234276	0.9529364733705119	1.0196104237629073	T	T	T
1.0027104126508233	0.4928698924695601	0.1019029370623722	T	T	T
0.9797358174695279	0.0063096072182668	0.0980506546824822	T	T	T
0.5548488992416417	0.2494385110132525	0.9931200996952401	T	T	T
0.9529830001690996	0.7638247890123352	1.0112142122880650	T	T	T
0.6787674095805054	0.3621482513562200	0.0583323229734216	T	T	T
0.3764430440462397	0.1543322688258826	0.0667111623986370	T	T	T
0.4652869109176905	0.7488001535701292	0.9877616965958257	T	T	T
0.0424663849225984	0.2394818579659035	1.0037764416521102	T	T	T
0.3190711376966230	0.6435347649819780	0.0399996824263604	T	T	T
0.6251819015636443	0.8405790890986498	0.0639952777045363	T	T	T
0.0039487801598239	0.4964253698685948	0.0371913225993236	T	T	T
0.9944214731889757	0.0028547363953691	0.0338268053511988	T	T	T
0.0000000000000000	0.0000000000000000	0.8841600000000014	F	F	F
0.2500000000000000	0.0000000000000000	0.8841600000000014	F	F	F
0.5000000000000000	0.0000000000000000	0.8841600000000014	F	F	F
0.7500000000000000	0.0000000000000000	0.8841600000000014	F	F	F
0.2499854034910882	0.8889018635634756	0.8841600000000014	F	F	F
0.4999854034910882	0.8889018635634756	0.8841600000000014	F	F	F
0.7499854034910882	0.8889018635634756	0.8841600000000014	F	F	F
0.9999854034910882	0.8889018635634756	0.8841600000000014	F	F	F
0.4999708069821835	0.7778037271269511	0.8841600000000014	F	F	F
0.7499708069821835	0.7778037271269511	0.8841600000000014	F	F	F
0.9999708069821835	0.7778037271269511	0.8841600000000014	F	F	F
0.2499708069821835	0.7778037271269511	0.8841600000000014	F	F	F
0.4999562104732718	0.6667055906904267	0.8841600000000014	F	F	F
0.7499562104732718	0.6667055906904267	0.8841600000000014	F	F	F
0.9999562104732718	0.6667055906904267	0.8841600000000014	F	F	F
0.2499562104732718	0.6667055906904267	0.8841600000000014	F	F	F
0.7499416139643671	0.5556074542538951	0.8841600000000014	F	F	F
0.9999416139643671	0.5556074542538951	0.8841600000000014	F	F	F

0.2499416139643671	0.5556074542538951	0.8841600000000014	F	F	F
0.4999416139643671	0.5556074542538951	0.8841600000000014	F	F	F
0.9999270172048966	0.4445093180400903	0.8841600000000014	F	F	F
0.2499270172048966	0.4445093180400903	0.8841600000000014	F	F	F
0.4999270172048966	0.4445093180400903	0.8841600000000014	F	F	F
0.7499270172048966	0.4445093180400903	0.8841600000000014	F	F	F
0.9999124210467727	0.3334111812917584	0.8841600000000014	F	F	F
0.2499124210467727	0.3334111812917584	0.8841600000000014	F	F	F
0.4999124210467727	0.3334111812917584	0.8841600000000014	F	F	F
0.7499124210467727	0.3334111812917584	0.8841600000000014	F	F	F
0.2498978243875314	0.2223130449888657	0.8841600000000014	F	F	F
0.4998978243875314	0.2223130449888657	0.8841600000000014	F	F	F
0.7498978243875314	0.2223130449888657	0.8841600000000014	F	F	F
0.9998978243875314	0.2223130449888657	0.8841600000000014	F	F	F
0.2498832277282830	0.1112149086859660	0.8841600000000014	F	F	F
0.4998832277282830	0.1112149086859660	0.8841600000000014	F	F	F
0.7498832277282830	0.1112149086859660	0.8841600000000014	F	F	F
0.9998832277282830	0.1112149086859660	0.8841600000000014	F	F	F
0.9165924276169264	0.9630289532293972	0.7899999999999991	F	F	F
0.1665924276169264	0.9630289532293972	0.7899999999999991	F	F	F
0.4165924276169264	0.9630289532293972	0.7899999999999991	F	F	F
0.6665924276169264	0.9630289532293972	0.7899999999999991	F	F	F
0.1668708240534542	0.8516703786191542	0.7899999999999991	F	F	F
0.4168708240534542	0.8516703786191542	0.7899999999999991	F	F	F
0.6668708240534542	0.8516703786191542	0.7899999999999991	F	F	F
0.9168708240534542	0.8516703786191542	0.7899999999999991	F	F	F
0.4166481069042334	0.7407572383073528	0.7899999999999991	F	F	F
0.6666481069042334	0.7407572383073528	0.7899999999999991	F	F	F
0.9166481069042334	0.7407572383073528	0.7899999999999991	F	F	F
0.1666481069042334	0.7407572383073528	0.7899999999999991	F	F	F
0.4164253897550125	0.6298440979955444	0.7899999999999991	F	F	F
0.6664253897550125	0.6298440979955444	0.7899999999999991	F	F	F
0.9164253897550125	0.6298440979955444	0.7899999999999991	F	F	F
0.1664253897550125	0.6298440979955444	0.7899999999999991	F	F	F
0.6667037861915333	0.5184855233853014	0.7899999999999991	F	F	F
0.9167037861915333	0.5184855233853014	0.7899999999999991	F	F	F
0.1667037861915333	0.5184855233853014	0.7899999999999991	F	F	F
0.4167037861915333	0.5184855233853014	0.7899999999999991	F	F	F
0.9164810690423195	0.4075723830735001	0.7899999999999991	F	F	F
0.1664810690423195	0.4075723830735001	0.7899999999999991	F	F	F
0.4164810690423195	0.4075723830735001	0.7899999999999991	F	F	F
0.6664810690423195	0.4075723830735001	0.7899999999999991	F	F	F
0.9167594654788402	0.2962138084632500	0.7899999999999991	F	F	F
0.1667594654788402	0.2962138084632500	0.7899999999999991	F	F	F
0.4167594654788402	0.2962138084632500	0.7899999999999991	F	F	F
0.6667594654788402	0.2962138084632500	0.7899999999999991	F	F	F
0.1665367483296194	0.1853006681514486	0.7899999999999991	F	F	F
0.4165367483296194	0.1853006681514486	0.7899999999999991	F	F	F
0.6665367483296194	0.1853006681514486	0.7899999999999991	F	F	F
0.9165367483296194	0.1853006681514486	0.7899999999999991	F	F	F
0.1663140311803986	0.0743875278396402	0.7899999999999991	F	F	F
0.4163140311803986	0.0743875278396402	0.7899999999999991	F	F	F
0.6663140311803986	0.0743875278396402	0.7899999999999991	F	F	F
0.9163140311803986	0.0743875278396402	0.7899999999999991	F	F	F
0.0831848552338528	0.9260579064587944	0.6959999999999980	F	F	F

0.3331848552338528	0.9260579064587944	0.6959999999999980	F	F	F
0.5831848552338528	0.9260579064587944	0.6959999999999980	F	F	F
0.8331848552338528	0.9260579064587944	0.6959999999999980	F	F	F
0.3334632516703806	0.8146993318485514	0.6959999999999980	F	F	F
0.5834632516703806	0.8146993318485514	0.6959999999999980	F	F	F
0.8334632516703806	0.8146993318485514	0.6959999999999980	F	F	F
0.0834632516703806	0.8146993318485514	0.6959999999999980	F	F	F
0.5832405345211598	0.7037861915367500	0.6959999999999980	F	F	F
0.8332405345211598	0.7037861915367500	0.6959999999999980	F	F	F
0.0832405345211598	0.7037861915367500	0.6959999999999980	F	F	F
0.3332405345211598	0.7037861915367500	0.6959999999999980	F	F	F
0.5835189309576805	0.5924276169264999	0.6959999999999980	F	F	F
0.8335189309576805	0.5924276169264999	0.6959999999999980	F	F	F
0.0835189309576805	0.5924276169264999	0.6959999999999980	F	F	F
0.3335189309576805	0.5924276169264999	0.6959999999999980	F	F	F
0.8332962138084667	0.4815144766146986	0.6959999999999980	F	F	F
0.0832962138084667	0.4815144766146986	0.6959999999999980	F	F	F
0.3332962138084667	0.4815144766146986	0.6959999999999980	F	F	F
0.5832962138084667	0.4815144766146986	0.6959999999999980	F	F	F
0.0830734966592459	0.3706013363028973	0.6959999999999980	F	F	F
0.3330734966592459	0.3706013363028973	0.6959999999999980	F	F	F
0.5830734966592459	0.3706013363028973	0.6959999999999980	F	F	F
0.8330734966592459	0.3706013363028973	0.6959999999999980	F	F	F
0.0833518930957666	0.2592427616926472	0.6959999999999980	F	F	F
0.3333518930957666	0.2592427616926472	0.6959999999999980	F	F	F
0.5833518930957666	0.2592427616926472	0.6959999999999980	F	F	F
0.8333518930957666	0.2592427616926472	0.6959999999999980	F	F	F
0.3331291759465458	0.1483296213808458	0.6959999999999980	F	F	F
0.5831291759465458	0.1483296213808458	0.6959999999999980	F	F	F
0.8331291759465458	0.1483296213808458	0.6959999999999980	F	F	F
0.0831291759465458	0.1483296213808458	0.6959999999999980	F	F	F
0.3334075723830736	0.0369710467706028	0.6959999999999980	F	F	F
0.5834075723830736	0.0369710467706028	0.6959999999999980	F	F	F
0.8334075723830736	0.0369710467706028	0.6959999999999980	F	F	F
0.0834075723830736	0.0369710467706028	0.6959999999999980	F	F	F

Supplementary Note 2. Atomic coordinates of the optimized structure of phase II film of VOTTDPz molecules after the structural optimization with VASP. The coordinates of gold substrate is included. The coordinates are expressed with a POSCAR/CONTCAR format used in VASP calculation.

```
project
   1.00000000000000
     5.7599999999999998  -39.9059999999999988    0.0000000000000000
    11.5199999999999996    0.0000000000000000    0.0000000000000000
     0.0000000000000000    0.0000000000000000   30.0000000000000000
   C    N    O    S    V    Au
    64   64    4   16    4  192
Selective dynamics
Direct
  0.9312121232374864  0.1079389316372712  0.3270025270360293   T   T   T
  0.9675245493200535  0.2417901954451196  0.3181423502286052   T   T   T
  0.9121391584332201  0.2141083911705266  0.3093454201882437   T   T   T
  0.0740688818021269  0.8899642837935602  0.3295183305256231   T   T   T
  0.9243630684295567  0.7983572566258488  0.3539474568898413   T   T   T
  0.9368859997752494  0.9075580091781816  0.3437695687267919   T   T   T
  0.9347410262766687  0.2971105555930151  0.3027636262684723   T   T   T
  0.0243475706643110  0.2388484784724412  0.3346352729312869   T   T   T
  0.9514276919808751  0.7062052345154513  0.3472719622557747   T   T   T
  0.0534270325799184  0.2931184614042905  0.3474738322948596   T   T   T
  0.0806357500367909  0.2009869374838402  0.3538574051696628   T   T   T
  0.0682526201003100  0.0915528841508504  0.3438090573495529   T   T   T
  0.9806282901993413  0.7601282423364637  0.3351053772708568   T   T   T
  0.4541923693776866  0.1945409556162971  0.3142357193425767   T   T   T
  0.4204180943000964  0.8331836203292724  0.2860069088245538   T   T   T
  0.4559860025316382  0.8276027178649983  0.2941210676223776   T   T   T
  0.5104458085050609  0.7256440215028590  0.2942706963726991   T   T   T
  0.5341269283320429  0.6199333229351822  0.2890011602627851   T   T   T
  0.5667709269036934  0.6503131941157605  0.2942221799033362   T   T   T
  0.5061308378097138  0.2441446931957785  0.3270956564575442   T   T   T
  0.4497914058811858  0.3187992400507724  0.3248771672162789   T   T   T
  0.5627642926176080  0.7767572374517826  0.3001128162173217   T   T   T
  0.4062477738514758  0.9536294521083803  0.2873452883959686   T   T   T
  0.4332300542752776  0.0199279122098162  0.2982791445980624   T   T   T
  0.4819515896790776  0.3488136347989368  0.3347695825556585   T   T   T
  0.5606854013293869  0.1465544864911834  0.3152525171869911   T   T   T
  0.0374912830404597  0.7573647041672444  0.3201871386563511   T   T   T
  0.5956779427735697  0.1463436054514347  0.3028331118650414   T   T   T
  0.6093965040002942  0.0276878845443349  0.2943888771977612   T   T   T
  0.5832083751399679  0.9563664365694535  0.3027483544879459   T   T   T
  0.0703114339877118  0.7005729141147157  0.3061518988642504   T   T   T
  0.3353277549751610  0.9432190149264770  0.3743392954669176   T   T   T
  0.3592901532195114  0.3115205434875961  0.4138959290639619   T   T   T
  0.3267972603209657  0.3133534511890375  0.3925182394055327   T   T   T
```

0.2745409947571318	0.4110210789223601	0.3689446395791478	T	T	T
0.2492951408749775	0.5143486614448420	0.3659691751950760	T	T	T
0.2215251552136195	0.4805886199990326	0.3427028809922348	T	T	T
0.2901219775454322	0.8884154966085092	0.3388681472920894	T	T	T
0.3403150973132583	0.8156968767194215	0.3726351254823541	T	T	T
0.2292674261039380	0.3561780529496588	0.3332998481559883	T	T	T
0.3749807197676915	0.1929865098218286	0.4145031603078664	T	T	T
0.3517874274003816	0.1227558085612230	0.3942339226656628	T	T	T
0.3125414755536440	0.7820485859010162	0.3494603105785927	T	T	T
0.2388501653440045	0.9868578628012585	0.3132067547910182	T	T	T
0.2050381009650053	0.9863660796444904	0.2971616552542002	T	T	T
0.1893878506715723	0.1047090100720567	0.2953107479121755	T	T	T
0.2138211371869190	0.1770697823167424	0.3105636896180499	T	T	T
0.0932892366763411	0.7814219013785220	0.3135866231234843	T	T	T
0.6788877562545395	0.0269381787489991	0.3798188034380132	T	T	T
0.6509261210889363	0.6597365132538613	0.4109014269885378	T	T	T
0.6851456702644327	0.6584929432590343	0.3946056137611009	T	T	T
0.7397291607176584	0.5658860638475289	0.3784047103082955	T	T	T
0.7663360502526686	0.4667098787049895	0.3809828493827396	T	T	T
0.7945986789885779	0.4998862069599639	0.3586198285878339	T	T	T
0.7249168165192614	0.0823148651093319	0.3467486263064004	T	T	T
0.6756321985485272	0.1536951627741132	0.3826030899634247	T	T	T
0.7856351288666801	0.6199263928961614	0.3435607243265082	T	T	T
0.6351371743030398	0.7782482140195128	0.4091189558520156	T	T	T
0.6598981072819399	0.8484245064274774	0.3934731148194643	T	T	T
0.7039028979748281	0.1882817766476848	0.3608462042861120	T	T	T
0.7750485401365879	0.9850075816994703	0.3177069775737849	T	T	T
0.8078989688039115	0.9852893927619206	0.2986013181721319	T	T	T
0.8238298999762534	0.8671701618159773	0.2962015920175745	T	T	T
0.8002689731929422	0.7951151364752889	0.3148355625085628	T	T	T
0.0869426316155568	0.9871691675192480	0.3394477059042700	T	T	T
0.1253679053361836	0.7406602497735619	0.3036942945066841	T	T	T
0.0841767113985199	0.5963933107928625	0.2895011402917262	T	T	T
0.9711203516558271	0.8812778298966890	0.3354458333845187	T	T	T
0.9182937390921211	0.0115587589825950	0.3381127673800819	T	T	T
0.9441095985938119	0.5978091307561115	0.3550175446042658	T	T	T
0.8954566431918707	0.7632399734683588	0.3669075214490150	T	T	T
0.8803357780042163	0.2506082199189166	0.2970404584828117	T	T	T
0.9205866585804969	0.3995418898331096	0.2848883255364001	T	T	T
0.9644817568226500	0.1264627049619094	0.3304257933124859	T	T	T
0.9944579812162715	0.2953014714293261	0.3230899403809246	T	T	T
0.0606080902140178	0.4011729068368766	0.3561314204874009	T	T	T
0.1094566014562375	0.2359788777483942	0.3672823525386235	T	T	T
0.0339942169200711	0.1176180998669309	0.3352607733471729	T	T	T
0.0106138374486306	0.7034310412320366	0.3244005272347081	T	T	T
0.4291076778931497	0.1354865488359351	0.3044527009756379	T	T	T
0.4774205233653248	0.7286704090388980	0.2918338096837587	T	T	T
0.5284716885630445	0.8186855721794615	0.3013289600305313	T	T	T
0.5878263655717078	0.8400563847888733	0.2996885628109638	T	T	T
0.5917466242481026	0.5595579556978336	0.2915404595328042	T	T	T
0.5329952943561196	0.5062258807737834	0.2807856507324118	T	T	T
0.4628359920976948	0.9411426239257707	0.3019100018722796	T	T	T
0.3989321549997413	0.7576788245058665	0.2765102435629743	T	T	T
0.3732897586840238	0.9744990064789434	0.2782029870536675	T	T	T
0.4242560289051625	0.4071740192431434	0.3286821930445925	T	T	T

0.4818703725837423	0.4608505830102771	0.3474050567476965	T	T	T
0.4883139493370408	0.1519566855573018	0.3171232908419768	T	T	T
0.5392123422617061	0.2420138583325340	0.3250086004204249	T	T	T
0.6168800355700341	0.2241445450798878	0.2945990883453433	T	T	T
0.6410524753270650	0.0108670494407761	0.2791705740942803	T	T	T
0.5540333593025721	0.0309696019557038	0.3136604704162096	T	T	T
0.3571414892996430	0.0063727882708733	0.3921112353400460	T	T	T
0.3045710545954989	0.4094502636837021	0.3875113324469055	T	T	T
0.2614882581768398	0.3173148426642882	0.3493437272756097	T	T	T
0.2076455586780952	0.2929587011879491	0.3150163486511204	T	T	T
0.1955891839269981	0.5666248346674010	0.3367046237930680	T	T	T
0.2451042027616097	0.6269790784319156	0.3793954239521682	T	T	T
0.3235262396949850	0.1985212966662004	0.3803579755764019	T	T	T
0.3773560180558420	0.3886178402104221	0.4323904947755963	T	T	T
0.4055849186833527	0.1753075053671367	0.4332362884251850	T	T	T
0.3634787977781073	0.7258859546158831	0.3854479523371168	T	T	T
0.3137974624640520	0.6660287136793315	0.3430001615738476	T	T	T
0.3048828183408716	0.9841403456857378	0.3535304618125465	T	T	T
0.2602266713149106	0.8896299642106769	0.3200988578941164	T	T	T
0.1852615455747824	0.9059153002923068	0.2866304257095891	T	T	T
0.1569528508642861	0.1188363439598916	0.2829998948015131	T	T	T
0.2433437610058036	0.1028837839322634	0.3211616657896670	T	T	T
0.0405832374710755	0.8728566038255465	0.3321105393711150	T	T	T
0.6553899623431008	0.9649176100277472	0.3934046038886265	T	T	T
0.7086653039325057	0.5649115655125883	0.3935006783883992	T	T	T
0.7525238095802038	0.6572336527910636	0.3566462872870488	T	T	T
0.8068100258240563	0.6804199615780107	0.3226537440417871	T	T	T
0.8218944709203697	0.4178907910303948	0.3580868408179896	T	T	T
0.7713965707683244	0.3583367422044859	0.3987732740402663	T	T	T
0.6890551129294806	0.7730939638449712	0.3831233235727112	T	T	T
0.6312234508793679	0.5839016893265949	0.4265723310896448	T	T	T
0.6031066050827008	0.7965634474887864	0.4228257286885793	T	T	T
0.6536551957456211	0.2424428159383183	0.3987745953172137	T	T	T
0.7043220783161104	0.3045063629798008	0.3590404229515300	T	T	T
0.7092877542442508	0.9862542443469167	0.3587992251469032	T	T	T
0.7542368742297398	0.0823611187607511	0.3266891667731642	T	T	T
0.8263418640498656	0.0670656398414309	0.2851769405093805	T	T	T
0.8553860529985826	0.8551221411284118	0.2802765866345851	T	T	T
0.7709859874443410	0.8692978299424752	0.3265694572406161	T	T	T
0.0015792032997766	0.0029874658769842	0.4055879729330840	T	T	T
0.5071831461428431	0.9586257045913840	0.3795532761782354	T	T	T
0.3037365417404203	0.1736752604009268	0.2857805690160191	T	T	T
0.7106325639491331	0.7991257611919522	0.2901860984834954	T	T	T
0.1243599535756061	0.6062727201789428	0.2851038308379827	T	T	T
0.9043230103707387	0.6192761135081284	0.3694912371853576	T	T	T
0.8808194220607461	0.3843575192296242	0.2780473905505687	T	T	T
0.1003715293448195	0.3797743547895109	0.3709476845008837	T	T	T
0.5722423514952908	0.4450667386214064	0.2820357979047046	T	T	T
0.3629742526760751	0.8432587252858355	0.2681168158264384	T	T	T
0.4423703068529719	0.5198783104918974	0.3447021656940379	T	T	T
0.6512466028087829	0.1441355489533009	0.2760511105334587	T	T	T
0.2078591145275936	0.6820662569676799	0.3606762098092773	T	T	T
0.4123111466598601	0.3073395632507996	0.4484503356438907	T	T	T
0.3487589601521179	0.6082044597858953	0.3672885849446388	T	T	T
0.1488946391777191	0.9840299374912007	0.2751526262359292	T	T	T

0.8101896015433141	0.3062778800443766	0.3853082235242411	T	T	T
0.5949433139220872	0.6655708745873241	0.4368535655965289	T	T	T
0.6700045688440639	0.3610677678101055	0.3853710918638100	T	T	T
0.8622203830244715	0.9907627203025910	0.2705327540702029	T	T	T
0.0023288126262813	0.0003378127915141	0.3517608873122313	T	T	T
0.5081040958978704	0.9789633083034275	0.3264188129057644	T	T	T
0.2886578699265314	0.1562055718993491	0.3347695396794208	T	T	T
0.7253027952291617	0.8158000594880406	0.3394637853190545	T	T	T
0.0000000000000000	0.0000000000000000	0.1701333333333324	F	F	F
0.0000000000000000	0.2500000000000000	0.1701333333333324	F	F	F
0.0000000000000000	0.5000000000000000	0.1701333333333324	F	F	F
0.0000000000000000	0.7500000000000000	0.1701333333333324	F	F	F
0.0625007057334770	0.0937496471332651	0.1701333333333324	F	F	F
0.0625007057334770	0.3437496471332651	0.1701333333333324	F	F	F
0.0625007057334770	0.5937496471332651	0.1701333333333324	F	F	F
0.0625007057334770	0.8437496471332651	0.1701333333333324	F	F	F
0.1250014114669469	0.1874992942665230	0.1701333333333324	F	F	F
0.1250014114669469	0.4374992942665230	0.1701333333333324	F	F	F
0.1250014114669469	0.6874992942665230	0.1701333333333324	F	F	F
0.1250014114669469	0.9374992942665230	0.1701333333333324	F	F	F
0.1875021172004239	0.0312489413997881	0.1701333333333324	F	F	F
0.1875021172004239	0.2812489413997881	0.1701333333333324	F	F	F
0.1875021172004239	0.5312489413997881	0.1701333333333324	F	F	F
0.1875021172004239	0.7812489413997881	0.1701333333333324	F	F	F
0.2500028229338938	0.1249985885330531	0.1701333333333324	F	F	F
0.2500028229338938	0.3749985885330531	0.1701333333333324	F	F	F
0.2500028229338938	0.6249985885330531	0.1701333333333324	F	F	F
0.2500028229338938	0.8749985885330531	0.1701333333333324	F	F	F
0.3125035285420736	0.2187482357289596	0.1701333333333324	F	F	F
0.3125035285420736	0.4687482357289667	0.1701333333333324	F	F	F
0.3125035285420736	0.7187482357289667	0.1701333333333324	F	F	F
0.3125035285420736	0.9687482357289667	0.1701333333333324	F	F	F
0.3750042344509623	0.0624978827745224	0.1701333333333324	F	F	F
0.3750042344509623	0.3124978827745224	0.1701333333333324	F	F	F
0.3750042344509623	0.5624978827745224	0.1701333333333324	F	F	F
0.3750042344509623	0.8124978827745224	0.1701333333333324	F	F	F
0.4375049401092568	0.1562475299453752	0.1701333333333324	F	F	F
0.4375049401092568	0.4062475299453752	0.1701333333333324	F	F	F
0.4375049401092568	0.6562475299453752	0.1701333333333324	F	F	F
0.4375049401092568	0.9062475299453752	0.1701333333333324	F	F	F
0.5000056457675512	0.9999971771162208	0.1701333333333324	F	F	F
0.5000056457675512	0.2499971771162208	0.1701333333333324	F	F	F
0.5000056457675512	0.4999971771162208	0.1701333333333324	F	F	F
0.5000056457675512	0.7499971771162208	0.1701333333333324	F	F	F
0.5625063516764399	0.0937468241617836	0.1701333333333324	F	F	F
0.5625063516764399	0.3437468241617836	0.1701333333333324	F	F	F
0.5625063516764399	0.5937468241617836	0.1701333333333324	F	F	F
0.5625063516764399	0.8437468241617836	0.1701333333333324	F	F	F
0.6250070573347344	0.1874964713326293	0.1701333333333324	F	F	F
0.6250070573347344	0.4374964713326293	0.1701333333333324	F	F	F
0.6250070573347344	0.6874964713326293	0.1701333333333324	F	F	F
0.6250070573347344	0.9374964713326293	0.1701333333333324	F	F	F
0.6875077629930360	0.0312461185034820	0.1701333333333324	F	F	F
0.6875077629930360	0.2812461185034820	0.1701333333333324	F	F	F
0.6875077629930360	0.5312461185034820	0.1701333333333324	F	F	F

0.6875077629930360	0.7812461185034820	0.1701333333333324	F	F	F
0.7500084686513304	0.1249957656743348	0.1701333333333324	F	F	F
0.7500084686513304	0.3749957656743348	0.1701333333333324	F	F	F
0.7500084686513304	0.6249957656743348	0.1701333333333324	F	F	F
0.7500084686513304	0.8749957656743348	0.1701333333333324	F	F	F
0.8125091745602191	0.2187454127198905	0.1701333333333324	F	F	F
0.8125091745602191	0.4687454127198905	0.1701333333333324	F	F	F
0.8125091745602191	0.7187454127198905	0.1701333333333324	F	F	F
0.8125091745602191	0.9687454127198905	0.1701333333333324	F	F	F
0.8750098802185136	0.0624950598907432	0.1701333333333324	F	F	F
0.8750098802185136	0.3124950598907432	0.1701333333333324	F	F	F
0.8750098802185136	0.5624950598907432	0.1701333333333324	F	F	F
0.8750098802185136	0.8124950598907432	0.1701333333333324	F	F	F
0.9375105858768080	0.1562447070615960	0.1701333333333324	F	F	F
0.9375105858768080	0.4062447070615960	0.1701333333333324	F	F	F
0.9375105858768080	0.6562447070615960	0.1701333333333324	F	F	F
0.9375105858768080	0.9062447070615960	0.1701333333333324	F	F	F
0.0208335685861769	0.8645832157069080	0.0917999999999992	F	F	F
0.0208335685861769	0.1145832157069080	0.0917999999999992	F	F	F
0.0208335685861769	0.3645832157069080	0.0917999999999992	F	F	F
0.0208335685861769	0.6145832157069080	0.0917999999999992	F	F	F
0.0833342743196539	0.9583328628401730	0.0917999999999992	F	F	F
0.0833342743196539	0.2083328628401730	0.0917999999999992	F	F	F
0.0833342743196539	0.4583328628401730	0.0917999999999992	F	F	F
0.0833342743196539	0.7083328628401730	0.0917999999999992	F	F	F
0.1458349800280629	0.0520825099859650	0.0917999999999992	F	F	F
0.1458349800280629	0.3020825099859650	0.0917999999999992	F	F	F
0.1458349800280629	0.5520825099859650	0.0917999999999992	F	F	F
0.1458349800280629	0.8020825099859650	0.0917999999999992	F	F	F
0.2083356857615399	0.8958321571192300	0.0917999999999992	F	F	F
0.2083356857615399	0.1458321571192300	0.0917999999999992	F	F	F
0.2083356857615399	0.3958321571192300	0.0917999999999992	F	F	F
0.2083356857615399	0.6458321571192300	0.0917999999999992	F	F	F
0.2708363915200707	0.9895818042399611	0.0917999999999992	F	F	F
0.2708363915200707	0.2395818042399611	0.0917999999999992	F	F	F
0.2708363915200707	0.4895818042399611	0.0917999999999992	F	F	F
0.2708363915200707	0.7395818042399611	0.0917999999999992	F	F	F
0.3333370971783722	0.0833314514108139	0.0917999999999992	F	F	F
0.3333370971783722	0.3333314514108139	0.0917999999999992	F	F	F
0.3333370971783722	0.5833314514108139	0.0917999999999992	F	F	F
0.3333370971783722	0.8333314514108139	0.0917999999999992	F	F	F
0.3958378030872538	0.9270810984563695	0.0917999999999992	F	F	F
0.3958378030872538	0.1770810984563695	0.0917999999999992	F	F	F
0.3958378030872538	0.4270810984563695	0.0917999999999992	F	F	F
0.3958378030872538	0.6770810984563695	0.0917999999999992	F	F	F
0.4583385087455554	0.0208307456272223	0.0917999999999992	F	F	F
0.4583385087455554	0.2708307456272223	0.0917999999999992	F	F	F
0.4583385087455554	0.5208307456272223	0.0917999999999992	F	F	F
0.4583385087455554	0.7708307456272223	0.0917999999999992	F	F	F
0.5208392144038498	0.8645803927980751	0.0917999999999992	F	F	F
0.5208392144038498	0.1145803927980751	0.0917999999999992	F	F	F
0.5208392144038498	0.3645803927980751	0.0917999999999992	F	F	F
0.5208392144038498	0.6145803927980751	0.0917999999999992	F	F	F
0.5833399200621443	0.9583300399689278	0.0917999999999992	F	F	F
0.5833399200621443	0.2083300399689278	0.0917999999999992	F	F	F

0.5833399200621443	0.4583300399689278	0.0917999999999992	F	F	F
0.5833399200621443	0.7083300399689278	0.0917999999999992	F	F	F
0.6458406259710330	0.0520796870144835	0.0917999999999992	F	F	F
0.6458406259710330	0.3020796870144835	0.0917999999999992	F	F	F
0.6458406259710330	0.5520796870144835	0.0917999999999992	F	F	F
0.6458406259710330	0.8020796870144835	0.0917999999999992	F	F	F
0.7083413316293274	0.8958293341853363	0.0917999999999992	F	F	F
0.7083413316293274	0.1458293341853363	0.0917999999999992	F	F	F
0.7083413316293274	0.3958293341853363	0.0917999999999992	F	F	F
0.7083413316293274	0.6458293341853363	0.0917999999999992	F	F	F
0.7708420372876290	0.9895789813561890	0.0917999999999992	F	F	F
0.7708420372876290	0.2395789813561890	0.0917999999999992	F	F	F
0.7708420372876290	0.4895789813561890	0.0917999999999992	F	F	F
0.7708420372876290	0.7395789813561890	0.0917999999999992	F	F	F
0.8333427431965106	0.0833286284017447	0.0917999999999992	F	F	F
0.8333427431965106	0.3333286284017447	0.0917999999999992	F	F	F
0.8333427431965106	0.5833286284017447	0.0917999999999992	F	F	F
0.8333427431965106	0.8333286284017447	0.0917999999999992	F	F	F
0.8958434488548122	0.9270782755725975	0.0917999999999992	F	F	F
0.8958434488548122	0.1770782755725975	0.0917999999999992	F	F	F
0.8958434488548122	0.4270782755725975	0.0917999999999992	F	F	F
0.8958434488548122	0.6770782755725975	0.0917999999999992	F	F	F
0.9583441545131066	0.0208279227434502	0.0917999999999992	F	F	F
0.9583441545131066	0.2708279227434502	0.0917999999999992	F	F	F
0.9583441545131066	0.5208279227434502	0.0917999999999992	F	F	F
0.9583441545131066	0.7708279227434502	0.0917999999999992	F	F	F
0.0416671371472930	0.9791664314263500	0.0134666666666661	F	F	F
0.0416671371472930	0.2291664314263500	0.0134666666666661	F	F	F
0.0416671371472930	0.4791664314263500	0.0134666666666661	F	F	F
0.0416671371472930	0.7291664314263500	0.0134666666666661	F	F	F
0.1041678428807700	0.0729160785596150	0.0134666666666661	F	F	F
0.1041678428807700	0.3229160785596150	0.0134666666666661	F	F	F
0.1041678428807700	0.5729160785596150	0.0134666666666661	F	F	F
0.1041678428807700	0.8229160785596150	0.0134666666666661	F	F	F
0.1666685486142470	0.1666657256928801	0.0134666666666661	F	F	F
0.1666685486142470	0.4166657256928801	0.0134666666666661	F	F	F
0.1666685486142470	0.6666657256928801	0.0134666666666661	F	F	F
0.1666685486142470	0.9166657256928801	0.0134666666666661	F	F	F
0.2291692543477168	0.0104153728261380	0.0134666666666661	F	F	F
0.2291692543477168	0.2604153728261380	0.0134666666666661	F	F	F
0.2291692543477168	0.5104153728261451	0.0134666666666661	F	F	F
0.2291692543477168	0.7604153728261451	0.0134666666666661	F	F	F
0.2916699601563693	0.1041650199218154	0.0134666666666661	F	F	F
0.2916699601563693	0.3541650199218154	0.0134666666666661	F	F	F
0.2916699601563693	0.6041650199218154	0.0134666666666661	F	F	F
0.2916699601563693	0.8541650199218154	0.0134666666666661	F	F	F
0.3541706658146637	0.1979146670926681	0.0134666666666661	F	F	F
0.3541706658146637	0.4479146670926681	0.0134666666666661	F	F	F
0.3541706658146637	0.6979146670926681	0.0134666666666661	F	F	F
0.3541706658146637	0.9479146670926681	0.0134666666666661	F	F	F
0.4166713714729582	0.0416643142635209	0.0134666666666661	F	F	F
0.4166713714729582	0.2916643142635209	0.0134666666666661	F	F	F
0.4166713714729582	0.5416643142635209	0.0134666666666661	F	F	F
0.4166713714729582	0.7916643142635209	0.0134666666666661	F	F	F
0.4791720773818469	0.1354139613090766	0.0134666666666661	F	F	F

0.4791720773818469	0.3854139613090766	0.0134666666666661	F	F	F
0.4791720773818469	0.6354139613090766	0.0134666666666661	F	F	F
0.4791720773818469	0.8854139613090766	0.0134666666666661	F	F	F
0.5416727830401413	0.9791636084799293	0.0134666666666661	F	F	F
0.5416727830401413	0.2291636084799293	0.0134666666666661	F	F	F
0.5416727830401413	0.4791636084799293	0.0134666666666661	F	F	F
0.5416727830401413	0.7291636084799293	0.0134666666666661	F	F	F
0.6041734886984429	0.0729132556507821	0.0134666666666661	F	F	F
0.6041734886984429	0.3229132556507821	0.0134666666666661	F	F	F
0.6041734886984429	0.5729132556507821	0.0134666666666661	F	F	F
0.6041734886984429	0.8229132556507821	0.0134666666666661	F	F	F
0.6666741943567374	0.1666629028216278	0.0134666666666661	F	F	F
0.6666741943567374	0.4166629028216278	0.0134666666666661	F	F	F
0.6666741943567374	0.6666629028216278	0.0134666666666661	F	F	F
0.6666741943567374	0.9166629028216278	0.0134666666666661	F	F	F
0.7291749002656260	0.0104125498671905	0.0134666666666661	F	F	F
0.7291749002656260	0.2604125498671905	0.0134666666666661	F	F	F
0.7291749002656260	0.5104125498671905	0.0134666666666661	F	F	F
0.7291749002656260	0.7604125498671905	0.0134666666666661	F	F	F
0.7916756059239205	0.1041621970380362	0.0134666666666661	F	F	F
0.7916756059239205	0.3541621970380362	0.0134666666666661	F	F	F
0.7916756059239205	0.6041621970380362	0.0134666666666661	F	F	F
0.7916756059239205	0.8541621970380362	0.0134666666666661	F	F	F
0.8541763115822150	0.1979118442088890	0.0134666666666661	F	F	F
0.8541763115822150	0.4479118442088890	0.0134666666666661	F	F	F
0.8541763115822150	0.6979118442088890	0.0134666666666661	F	F	F
0.8541763115822150	0.9479118442088890	0.0134666666666661	F	F	F
0.9166770174911036	0.0416614912544446	0.0134666666666661	F	F	F
0.9166770174911036	0.2916614912544446	0.0134666666666661	F	F	F
0.9166770174911036	0.5416614912544446	0.0134666666666661	F	F	F
0.9166770174911036	0.7916614912544446	0.0134666666666661	F	F	F
0.9791777231493981	0.1354111384252974	0.0134666666666661	F	F	F
0.9791777231493981	0.3854111384252974	0.0134666666666661	F	F	F
0.9791777231493981	0.6354111384252974	0.0134666666666661	F	F	F
0.9791777231493981	0.8854111384252974	0.0134666666666661	F	F	F

Supplementary Note 3

Bulk structure deduced from XRD measurements in comparison with the film structure.

We examine the structure of the bulk crystal in order to compare with that of the film. In Figure 1, we show a space filling view of the β phase VOTTDPz bulk crystal. The model is constructed from the crystallographic information file (CIF file) shown in the supplemental information of Ref. 1.

In this phase, VOTTDPz molecules are ordered with 2D pai interaction with neighboring molecules. Thus the centers of the molecules are distributed in a plane. As obvious from the top view, VO-up and VO-down molecules appear in an alternative manner. In addition it should be noticed that VO-up and VO-down molecules are tilted from the plane and show a buckled structure.

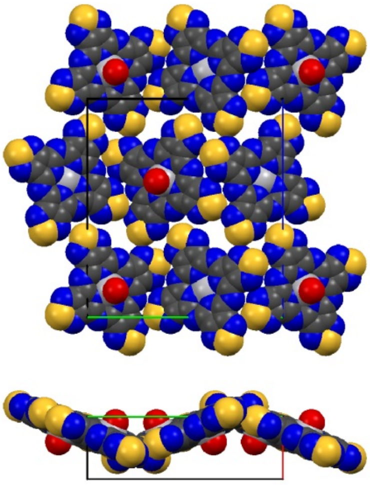

Supplementary Figure 1. A space filling view of the β phase VOTTDPz bulk crystal; top view (upper) and side view (lower). Light gray, dark gray, blue, yellow and red circles represent V, C, N, S and O atoms, respectively. Note that VO up and VO down molecules appear in an alternative manner.

Supplementary Note 4

XMCD measurement on VOPc and VOTTDPz

We compare the circular polarized x-ray absorption spectroscopy (XAS) obtained on the films of VO phthalocyanine (VOPc) and VOTTDPz molecules on Au(111). In each panel, XAS results for two circular directions and the XMCD plots are illustrated. The photon energy range includes V 2p and O 1s components. The XMCD intensity of the VOTTDPz component is doubled for the clarification. The XMCD intensity comparison between the VOPc film and the VOTTDPz film, by considering the doubled scale for the VOTTDPz film, indicates the magnetization is weaker for the VOTTDPz film.

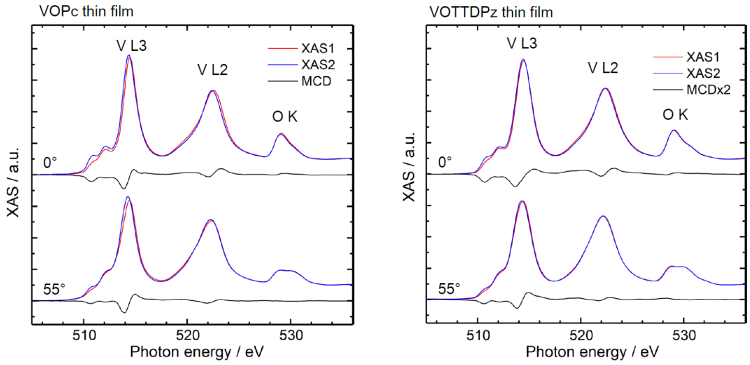

Supplementary Figure 2. Circular polarized XAS of films of VOPc (a), and TbPc2 (b) on Au(111) at T=5 K and B=5 T at 0° (normal) and 55o incidence (I+, I-) and XMCD for each case. Note XMCD plot for VOTTDPz is doubled in y-scale.

Supplementary References

1. Miyoshi, Y; Takahashi, K; Fujimoto, T; Yoshikawa, H; Matsushita, MM; Ouchi, Y; Kepenekian, M; Robert, V; Donzello, MP; Ercolani, C, et al. Crystal Structure, Spin Polarization, Solid-State Electrochemistry, and High N-Type Carrier Mobility of a Paramagnetic Semiconductor: Vanadyl Tetrakis(Thiadiazole)Porphyrazine. Inorg. Chem. 2012, 51, 456-462.
